# Supplementary material for: FlowPacker: protein side-chain packing with torsional flow matching
Source: Bioinformatics. 2025 Jan 9;41(3):btaf010. doi: 10.1093/bioinformatics/btaf010 (PMC11886813; doi:10.1093/bioinformatics/btaf010)
Supplement: btaf010_Supplementary_Data [file btaf010_supplementary_data.zip › 06bbc_FlowPacker_Appendix.pdf]

Supplementary Information

1.1 Performance issues with DiffPack.

The discrepancy in performance of DiffPack - as discussed in the main text - can be attributed to the way that it handles noising and denoising of the side-chain χ angles. We observed that DiffPack uses the ground-truth bond angles and bond lengths without idealization during training and inference [1], which is an issue due to two main reasons: 1. inference is not possible when the ground-truth side-chain coordinates are not known (ex. samples from backbone generative models), and 2. *a priori* information of input side-chain conformations should not impact predictive performance. The first reason is a trivial issue since any random initialization of torsion angles can be easily applied to backbone-only structures to serve as a starting point for side-chain packing tools. However, we discovered that for structures where ground-truth bond angles and bond lengths are unknown (i.e. using idealized structures), DiffPack performs notably worse, limiting applicability to side-chain packing for structures without access to ground-truth sidechain conformations.

First, we verify that the ground-truth bond angles and bond lengths are unchanged before and after DiffPack in Supplementary Figure 1. We analyze both Ca-Cβ bond length and N-Ca-Cβ bond angles for DiffPack and FlowPacker, and observe that DiffPack perfectly recapitulates the ground-truth distributions of both bond angles and bond lengths, while FlowPacker does not since it uses idealized bond angles and lengths.

To assess the performance discrepancy of using varying structures that **only differ in the side-chain coordinates**, we input various structures to both DiffPack and AttnPacker, one of the side-chain packing baselines used in this paper, and report the results in Supplementary Table 1. Note that in all the different input structures, the backbone coordinates are unchanged. First, we report that idealization of bond lengths and angles results in a $> 0.25A$ increase in RMSD, which provides significant advantage to DiffPack given that side-chain packing tools attains sub-Angstrom accuracy. Moreover, we observe that DiffPack shows worse performance when we use idealized, Rosetta-packed, and AttnPacker-packed structures as input, suggesting that the ground-truth bond angles and lengths contribute significantly to DiffPack's performance. On the other hand, AttnPacker shows the same performance across all inputs, since it is agnostic to the input sidechain coordinates. Therefore, we conclude that DiffPack does exhibit data leakage to some extent, since side-chain packing models should only be dependent on the backbone coordinates and not the input structure's side-chain coordinates. The current version of DiffPack may be useful when searching sidechain conformational states of known protein structures, but its applicability to structures without knowledge of the ground-truth side-chain conformations may be limited. We also note that DiffPack's performance may improve when retrained with idealized bond angles and lengths. We have contacted the authors of DiffPack to notify them of our findings.

1.2 Background

Preliminaries. Proteins are macromolecules that adopt three-dimensional conformations based on its amino acid identity and resulting covalent and non-covalent interactions between atoms. The atoms that constitute a full-atom protein structure are divided into backbone atoms, which are the N, Ca, C, and O atoms that constitute the peptide backbone, and side-chain atoms, which are dependent on the amino acid identity and can contain up to 9 heavy atoms in total. Side-chain flexibility is usually limited to four degrees of freedom defined by the four χ torsion angles whose empirical distributions are highly constrained based on amino acid type and neighboring atomic forces. The problem of side-chain packing, therefore, can be reduced to finding $\chi_{1...4} \in [0, 2\pi)$ conditioned on the protein sequence $s \in \{0, ..., 19\}$ and backbone coordinates $X_{bb} \in \mathcal{R}^3$.

Related Work. Side-chain packing methods has traditionally relied on energy-based sampling such as Rosetta [1] or SCWRL [2], but has largely been replaced with the rise of performant deep learning methods. Notably, AttnPacker [3] is a model based on AlphaFold2 [4] that uses sparse triangular attention and invariant point attention to directly predict all-atom coordinates. DiffPack [5] proposes a diffusion-based approach that applies a relational graph convolution network on atom-level

[1] see `rotate_side_chain` function in `https://github.com/DeepGraphLearning/DiffPack/blob/main/diffpack/rotamer.py`

graphs, with several innovations such as autoregressive sampling to attain state-of-the-art performance. SidechainDiff [6] presents a sidechain packing diffusion model for the prediction of mutational effects using transfer learning. More recently, two peptide-specific torsional flow matching models have been published, where PPFlow [7] uses torsional flow matching on both backbone and side-chain torsion angles for peptide design, while PepFlow [8] uses a multi-modal approach to full-atom peptide design against protein pockets, using $S(E)3$ flow matching for backbone generation, simplex flow for amino acid sequences, and torsional flow matching for side-chain χ angles.

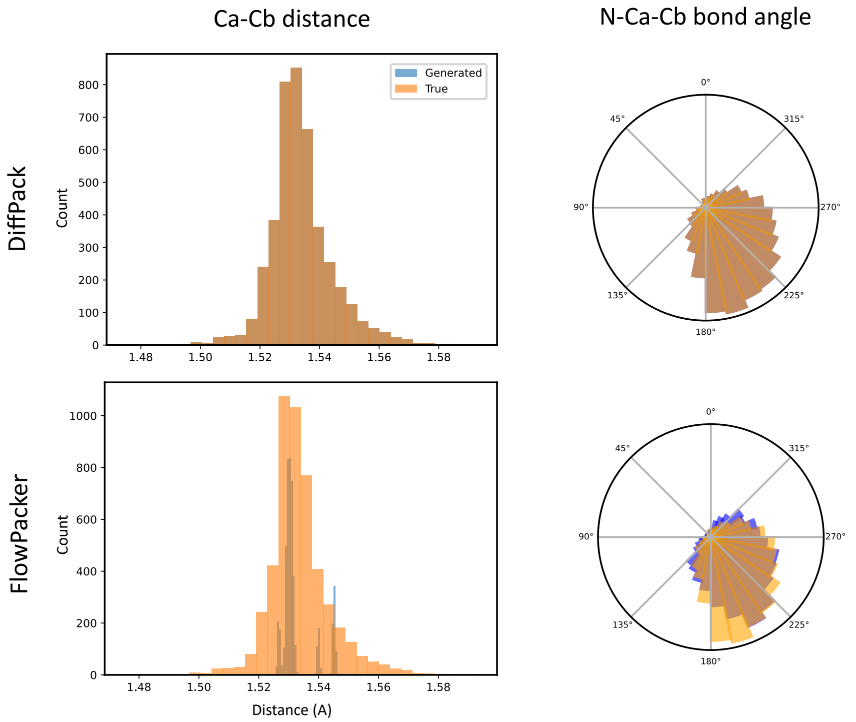

Figure S1: Analysis of N-Ca-Cβ bond angle and Ca-Cβ bond lengths before and after DiffPack and FlowPacker. We observe that DiffPack perfectly recapitulates the input structure's bond angle and lengths, which we believe may be a source of data leakage since it is not fully agnostic to the ground-truth sidechain. FlowPacker uses idealized coordinates given the noised torsions, which results in different distributions from the input ground-truth structures. Note that the variations in idealized distances of FlowPacker is a result of using ground-truth Ca coordinates with idealized Cβ ones. The plots were generated by using the CASP13 test set with the respective models.

		Angle MAE ° ↓				RMSD Å ↓
**Model**	**Input**	χ_1	χ_2	χ_3	χ_4	All
Idealizer	Ground Truth	1.33	0.02	0.03	0.03	0.254
DiffPack	Ground Truth	14.07	23.20	36.79	48.13	0.571
	Idealized	22.26	29.96	46.61	53.47	0.778
	Rosetta	21.43	29.90	46.20	54.25	0.771
	AttnPacker-pp	21.98	29.22	45.35	54.77	0.780
AttnPacker	Ground Truth	16.33	27.46	50.42	49.40	0.677
	Idealized	16.33	27.46	50.42	49.40	0.677
	Rosetta	16.33	27.46	50.42	49.40	0.677
	AttnPacker-pp	16.33	27.46	50.42	49.40	0.677

Table S1: Performance metrics of DiffPack and AttnPacker on various input structures on the CASP13 test set. We observe that idealization of bond angles and lengths results in a >0.25A RMSD from the ground-truth, suggesting models that directly use the ground-truth bond angles and lengths significantly benefits atom RMSD performance over idealized or agnostic packing tools.

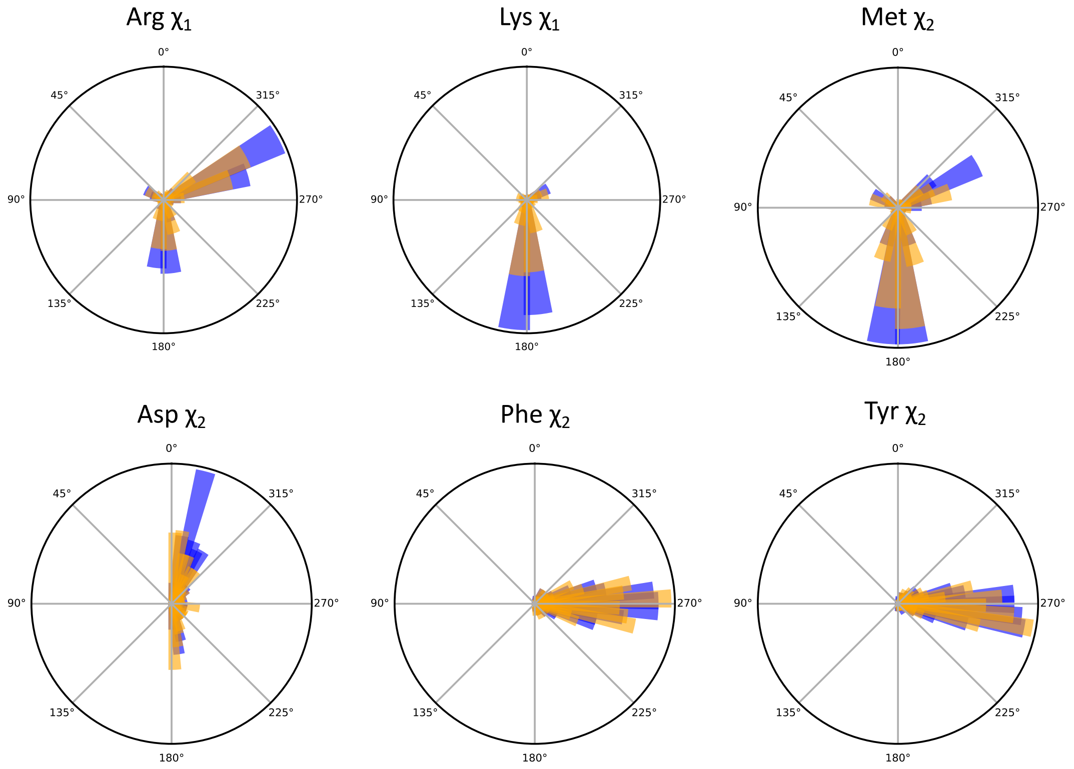

Figure S2: Generated vs. true χ distributions. Three randomly selected χ distributions are listed on the top row, while the bottom row contains three π-symmetric χ angles that lie in the interval $[0, 180]$ due to the parameterization in FlowPacker. Generated χ angles are depicted in orange, while ground-truth χ angles are in blue. The distributions are generated using the CASP13 test set.

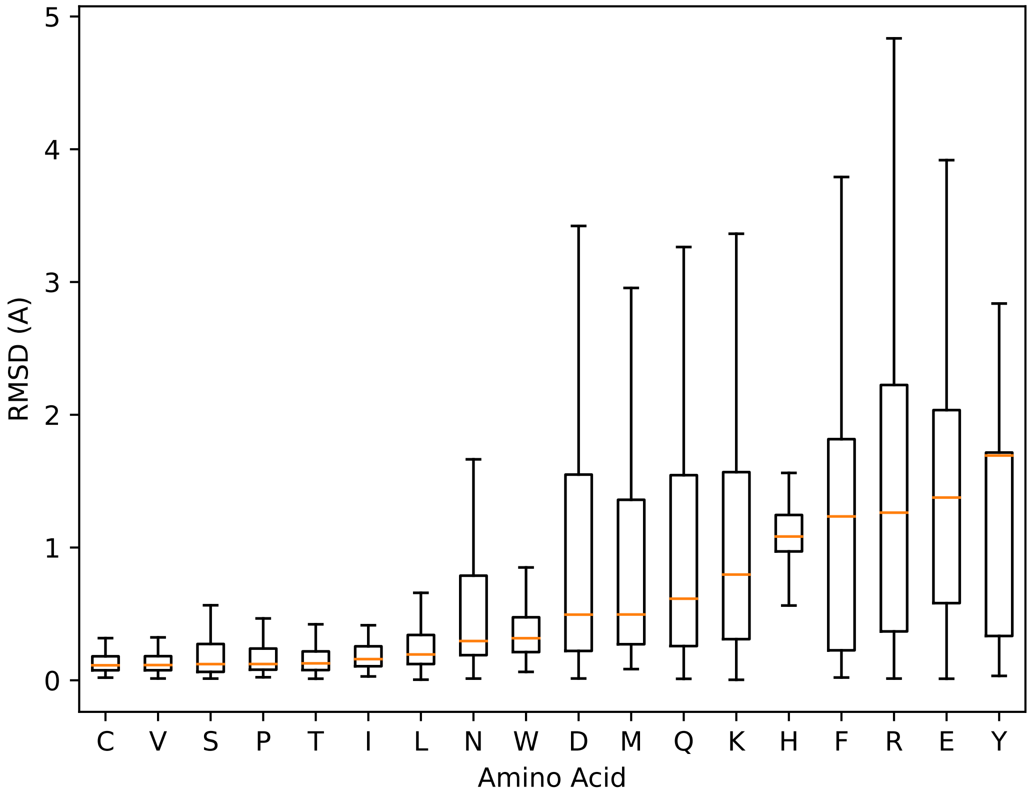

Figure S3: RMSD distributions per amino-acid identity, ranked from lowest to highest median atom RMSD. We observe that aromatic (Y, F, H) and long linear (E, R, K) sidechains exhibit the highest median RMSD.

Model	Clash Count		
	CASP13	CASP14	CASP15
Ground Truth	3.35	5.35	4.89
Rosetta	33.2	29.7	26.2
AttnPacker	56.2	63.8	46.5
DiffPack-fix	22.9	23.9	15.3
FlowPacker	**15.1**	**16.8**	**15.0**

Table S2: Number of clashes across different models and different datasets. Clashes are defined as if any atom pair is within 90% of the interatomic distance, where the measurements indicate the mean number of clashes per sample.

Dataset	Angle MAE ° ↓				Angle Accuracy % ↑				Atom RMSD Å ↓		
	χ_1	χ_2	χ_3	χ_4	χ_1	χ_2	χ_3	χ_4	All	Core	Surface
BC40	24.82	31.14	43.74	56.68	77.14%	67.46%	**48.90%**	45.61%	0.800	0.435	1.027
PDB-S40	**23.50**	**29.66**	**43.22**	**54.37**	**78.04%**	**68.71%**	48.69%	**46.42%**	**0.770**	**0.404**	**0.991**

Table S3: Performance evaluation of FlowPacker on CASP15 trained on two different datasets.

References

[1] R. F. Alford *et al.*, “The rosetta all-atom energy function for macromolecular modeling and design,” *Journal of chemical theory and computation*, vol. 13, no. 6, pp. 3031–3048, 2017.

[2] Q. Wang, A. A. Canutescu, and R. L. Dunbrack Jr, “Scwrl and molide: Computer programs for side-chain conformation prediction and homology modeling,” *Nature protocols*, vol. 3, no. 12, pp. 1832–1847, 2008.

[3] M. McPartlon and J. Xu, “An end-to-end deep learning method for protein side-chain packing and inverse folding,” *Proceedings of the National Academy of Sciences*, vol. 120, no. 23, e2216438120, 2023.

[4] J. Jumper *et al.*, “Highly accurate protein structure prediction with alphafold,” *Nature*, vol. 596, no. 7873, pp. 583–589, 2021.

[5] Y. Zhang, Z. Zhang, B. Zhong, S. Misra, and J. Tang, “Diffpack: A torsional diffusion model for autoregressive protein side-chain packing,” *Advances in Neural Information Processing Systems*, vol. 36, 2024.

[6] S. Liu, T. Zhu, M. Ren, C. Yu, D. Bu, and H. Zhang, “Predicting mutational effects on protein-protein binding via a side-chain diffusion probabilistic model,” *Advances in Neural Information Processing Systems*, vol. 36, 2024.

[7] H. Lin *et al.*, “Ppflow: Target-aware peptide design with torsional flow matching,” *bioRxiv*, pp. 2024–03, 2024.

[8] J. Li *et al.*, “Full-atom peptide design based on multi-modal flow matching,” *arXiv preprint arXiv:2406.00735*, 2024.
